# Supplementary material for: COZOID: contact zone identifier for visual analysis of protein-protein interactions
Source: BMC Bioinformatics. 2018 Apr 6;19:125. doi: 10.1186/s12859-018-2113-6 (PMC5889581; doi:10.1186/s12859-018-2113-6)
Supplement: Supplementary file 2 — Software build. Executable binary file of the software tool. (ZIP 118,784 kb) [file 12859_2018_2113_MOESM2_ESM.zip › cozoid/platform/docs/testng-6.5.1-javadoc.zip!/org/testng/annotations/Listeners.html]

Listeners (TestNG)


---


|  |  |  |
| --- | --- | --- |
| **Overview** \| **Package** \| **Class** \| **Tree** \| **Deprecated** \| **Index** \| **Help** | |  |
| **PREV CLASS**   **NEXT CLASS** | **FRAMES**    **NO FRAMES**     **All Classes** |  |
| SUMMARY: REQUIRED \| OPTIONAL | DETAIL: ELEMENT |  |


---


## org.testng.annotations Annotation Type Listeners

---

``` @Retention(value=RUNTIME) @Target(value=TYPE) public @interface Listeners ```

This annotation lets you define listeners directly on a test class
instead of doing so in your testng.xml. Any class that implements
the interface `ITestNGListener` is allowed,
except `IAnnotationTransformer` and `IAnnotationTransformer2`,
which need to be defined in XML since they have to be known before we even
start looking for annotations.
Note that listeners specified this way are global to your entire suite, just
like listeners specified in testng.xml.

**Author:**
:   Cedric Beust, Mar 26, 2010

---

| **Optional Element Summary** | |
| --- | --- |
| `java.lang.Class<? extends ITestNGListener>[]` | `value` |

### value

```
public abstract java.lang.Class<? extends ITestNGListener>[] value
```

:   **Default:**: {}


---


|  |  |  |
| --- | --- | --- |
| **Overview** \| **Package** \| **Class** \| **Tree** \| **Deprecated** \| **Index** \| **Help** | |  |
| **PREV CLASS**   **NEXT CLASS** | **FRAMES**    **NO FRAMES**     **All Classes** |  |
| SUMMARY: REQUIRED \| OPTIONAL | DETAIL: ELEMENT |  |


---
